# A novel type II collagen gene mutation in a family with spondyloepiphyseal dysplasia and extensive intrafamilial phenotypic diversity

**DOI:** 10.1038/hgv.2016.7

**Published:** 2016-05-19

**Authors:** Yasuharu Nakashima, Yuma Sakamoto, Gen Nishimura, Shiro Ikegawa, Yukihide Iwamoto

**Affiliations:** 1 Department of Orthopaedic Surgery, Graduate School of Medical Sciences, Kyushu University, Fukuoka, Japan; 2 Laboratory for Bone and Joint Diseases, RIKEN Center for Integrative Medical Sciences, Tokyo, Japan; 3 Department of Radiology, Tokyo Metropolitan Children’s Medical Center, Tokyo, Japan

## Abstract

The purpose of this study was to describe a family with spondyloepiphyseal dysplasia caused by a novel type II collagen gene (*COL2A1*) mutation and the family’s phenotypic diversity. Clinical and radiographic examinations of skeletal dysplasia were conducted on seven affected family members across two generations. The entire coding region of *COL2A1*, including the flanking intron regions, was analyzed with PCR and direct sequencing. The stature of the subjects ranged from extremely short to within normal height range. Hip deformity and advanced osteoarthritis were noted in all the subjects, ranging from severe coxa plana to mild acetabular dysplasia. Atlantoaxial subluxation combined with a hypoplastic odontoid process was found in three of the subjects. Various degrees of platyspondyly were confirmed in all subjects. Genetically, a novel *COL2A1* mutation (c.1349G>C, p.Gly450Ala) was identified in all the affected family members; however, it was not present in the one unaffected family member tested. We described a family with spondyloepiphyseal dysplasia and a novel *COL2A1* mutation (c.1349G>C, p.Gly450Ala). Phenotypes were diverse even among individuals with the same mutation and within the same family.

Disorders that arise from heterozygous mutations of *COL2A1* are collectively termed type II collagenopathies,^1^ and they are one of the major groups of skeletal dysplasias.^2^ More than 400 *COL2A1* mutations have been reported in the Human Gene Mutation Database (https://portal.biobase-international.com/hgmd/), and these mutations result in a diverse phenotypic spectrum that predominantly affects cartilage and bone.^3,4^ On the basis of clinical findings, type II collagenopathies are divided into several categories, including spondyloepiphyseal dysplasia (SED) spectrum, Stickler dysplasia type I and Kniest dysplasia.^5,6^

The SED spectrum is further divided into several phenotypes.^3^ Achondrogenesis type II and hypochondrogenesis are lethal and at the severe end of this spectrum. SED congenita is characterized by short stature with a short trunk and coxa vara. SED tarda indicates late-onset SED. The distinction among the SED spectrum phenotypes is mainly based on clinical features; however, considerable phenotypic diversity often hampers proper classification even with the same mutation.^5^

In the present study, we describe a Japanese family with SED caused by a novel *COL2A1* mutation. This mutation predominantly affected the hip joint and spine of the affected individuals in this family. In addition, extensive intrafamilial phenotypic diversity was observed.

This study was approved by the local Institutional Review Board, and informed consent was obtained from all participants.

The index case (case 4) was a 50-year-old female with bilateral hip pain during her initial examination. Plain radiographs showed joint space narrowing without acetabular dysplasia and atlantoaxial subluxation and platyspondyly in her thoracolumbar spine (Figure 1a–c). She was diagnosed with SED. Her family members also exhibited below average height, and/or spine and joint symptoms. Their pedigree is shown in Figure 2a. The patient’s skeletal problems seemed to be an inherited autosomal dominant trait; thus, we performed clinical and molecular surveys on seven affected members (cases 1–4 and 6–8) and one normal member (case 5) of her family.

Table 1 is a summary of clinical and radiographic findings from the family. The stature of affected members ranged from extremely short to the lower range of normal height (from −8.9 to −0.8 s.d.). The stature of case 5, who appeared normal, was +0.8 s.d. None of the affected members had any obvious cleft palate, hearing loss or visual impairments.

Hip joint deformity was confirmed in all affected individuals. Four individuals (cases 1, 2, 3 and 6) showed narrowing of joint spaces, some with and some without, and structural abnormalities of the acetabulum (Figure 1a). Cases 7 and 8 presented with severe deformities of the hip joints, high positions of the great trochanter, short necks and coxa plana. In contrast, deformities of the knee and ankle joints were not common except in case 8.

Three individuals exhibited atlantoaxial subluxation with hypoplasia of the odontoid process (Figure 1b). All affected individuals, except case 6, presented with various degrees of thoracolumbar platyspondyly. Case 8, who presented with the most severe phenotype, also showed spinal deformities, including vertebral hypoplasia and vertebral slippage (Figure 1b,c).

In the index case, we identified a novel missense mutation, c.1349G>C (the translation start site of NM_001844 was denoted as +1), in exon 21 of *COL2A1* (Figure 2b). This mutation was not listed in the Human Gene Mutation Database or in any single-nucleotide polymorphism database, including the dbSNP and the Human Genetic Variation browser. It was predicted to cause an amino-acid substitution from a glycine to an alanine at codon 450 (p.Gly450Ala) and to disrupt a well-regulated Gly–X–Y motif in the triple helical domain. This novel mutation was identified in all the other six affected individuals, but was not found in the one normal individual. Therefore, this mutation showed perfect co-segregation with the SED phenotype in this family.

In this report, we have described a family with a novel *COL2A1* mutation involving a Gly substitution in the triple helical domain: c.1349G>C, p.Gly450Ala. The phenotype of this family was SED, and none of the affected members had explicit visual or hearing impairments. Although Nishimura *et al.*^3^ indicated that Gly-to-nonserine substitutions often cause severe phenotypes, such as SED congenita with severe coxa vara or ocular disorders, this trend was not consistent in the individuals we describe here. They showed relatively mild phenotypes of SED, and there were no associated extraskeletal findings.

Including the newly identified mutation, 11 *COL2A1* mutations that lead to Gly-to-Ala substitutions in the triple helical domain have been reported.^3,7–13^ The phenotypes of these 11 mutations are extremely diverse. As both Gly and Ala are non-polar amino acids and their molecular weights are similar, the mild phenotype displayed by our index case could be expected from the Gly-to-Ala substitution. However, Gly-to-Ala substitutions in different positions cause lethal SED phenotypes such as achondrogenesis type II and hypochondrogenesis,^10,11^ suggesting that the position of the Gly-to-Ala substitution strongly affects the severity of the resulting phenotype.

Although the SED phenotype of this family was not lethal, there was extensive intrafamilial phenotypic diversity. Cases 7 and 8 displayed severe phenotypes associated with short statue, whereas other cases had milder phenotypes. We could not discover the mechanism underlying the phenotypic diversity in this family. It could be due to the additional genetic factors or environmental factors such as mechanical stress, as previous reports have mentioned.^5,7^

In conclusion, we described a family with SED caused by a novel *COL2A1* mutation (c.1349G>C, p.Gly450Ala). The resulting phenotypes were diverse even when caused by the same mutation and within the same family.

## Figures and Tables

**Figure 1:**
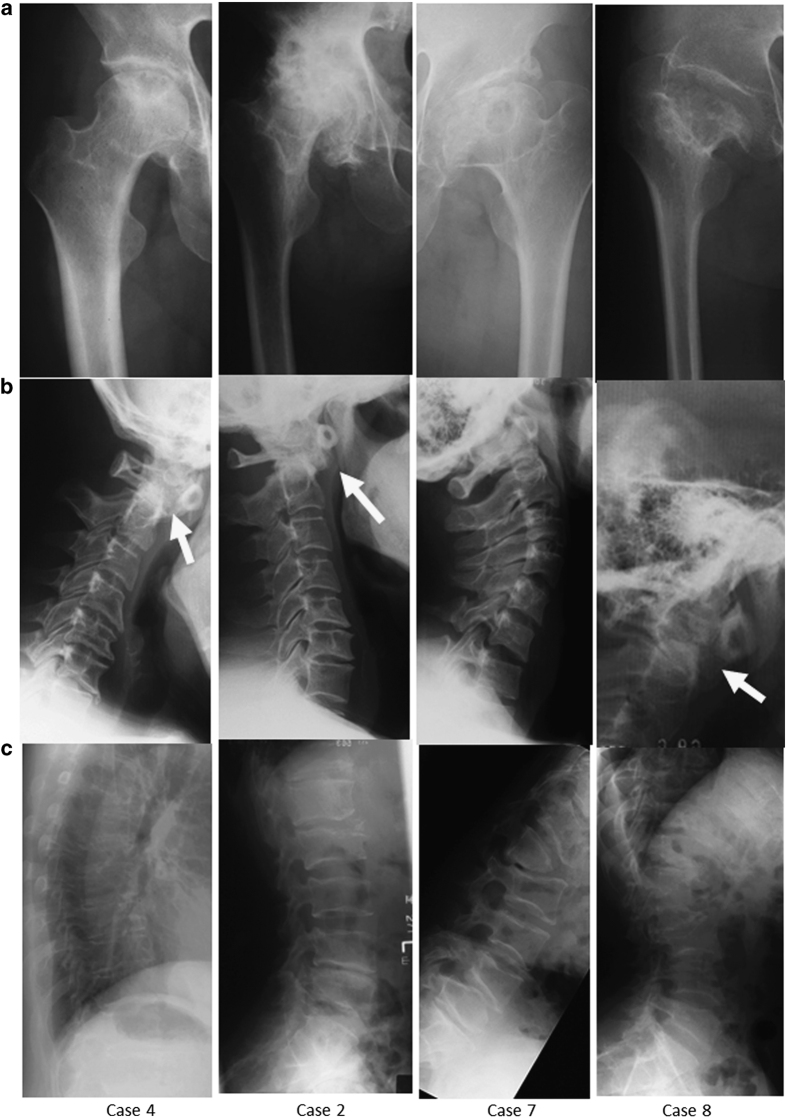
Radiographic findings of the family. (**a**) Hip joint: cystic and sclerotic changes in right femoral heads and the joint spaces were narrowed in case 4. Although structural deformation such as acetabular dysplasia was not common, joint spaces were narrowed in cases 2 and 7. In case 8, significant deformations such as high landing greater trochanter and coxa plana were confirmed. (**b**) Cervical spine: atlantoaxial subluxation with hypoplasia of odontoid process was confirmed in cases 4, 2 and 8, which was not confirmed in case 7. (**c**) Thracolumbar spine: although there are differences in the severity, platyspondyly was confirmed in all cases. In cases 7 and 8 with significant deformation, hypoplasia of the vertebral body and various slippages were confirmed.

**Figure 2:**
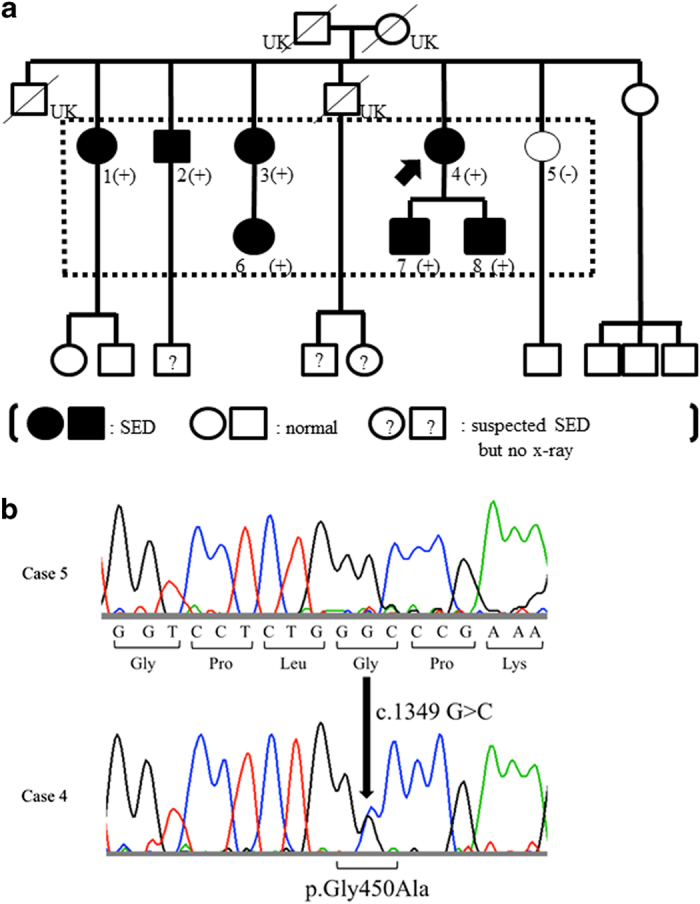
The pedigree of the family and co-segregation of the *COL2A1* mutation (**a**) and COL2A1 sequences (**b**). (**a**) The phenotype of SED was assumed to be inherited as an autosomal dominant trait. *COL2A1* mutation analysis was performed for the subjects surrounded by dotted lines, and all affected subjects had heterozygous c.1349G>C mutation in *COL2A1*. UK indicates that phenotype was unknown because subjects already passed away and no X-ray information. ?, suspected for SED but no radiographs available; SED, spondyloepiphyseal dysplasia. (**b**) DNA of each individual was extracted from saliva, and the entire coding regions of *COL2A1* (GenBank accession number: NM_001844.3) with flanking intronic regions were amplified by PCR. Case 5 showed normal sequence (top). On the other hand, case 4 showed heterozygous c.1349G>C mutation (bottom), which was predicted to result in Gly substitution at the Gly–X–Y motif.

**Table 1:** Clinical and radiographic findings of the family members

*Case*^a^	*Sex*	*Age*	*Height (cm)*	*s.d.*^b^	*Cervical spine*		*Thoracolumbar spine*		*Joints*		
					*Atlantoaxial subluxation*	*Other findings*	*Platyspondyly*	*Other findings*	*Hip deformation*	*Knee and ankle*	*Others*
1	F	84	136	−1.4	(−)		(+)		(+)	Mostly normal	(−)
2	M	82	ND	ND	(+)	Odontoid process hypoplasia	(+)		(+)	Mostly normal	(−)
3	F	81	140	−0.8	ND		(+)		(+)	Mostly normal	(−)
4	F	74	145	−0.9	(+)	Odontoid process hypoplasia	(+)		(+)	Mostly normal	Shoulder OA
5	F	71	155	+0.8	(−)		(−)		(−)	Normal	(−)
6	F	53	ND	ND	ND		ND		(+)	ND	ND
7	M	49	128	−7.5	(−)		(+)	L1 hypoplasia, L4 slippage	(+)	Mostly normal	ND
8	M	46	120	−8.9	(+)	Odontoid process hypoplasia	(+)	Scoliosis, L1, 2, 3 hypoplasia	(+)	Deformation in proximal tibia	Deformation in elbows

Abbreviations: F, female; M, male; ND, no data; OA, osteoarthritis.

a Correspond to patient ID in Figure 1.

b Based on the National Health and Nutrition Survey in Japan, 2013 (Ministry of Health, Labour and Welfare).
